# Anterior cervical abscess caused by migration of an ingested fish bone in a child: A case report

**DOI:** 10.1016/j.radcr.2026.08.016

**Published:** 2026-08-30

**Authors:** Ibrahima Faye, Cherif Mohamadou Aidara, Fallou Galass Niang, Saye Ngom, Oumou Diallo, Cheikh Ahmadou Bamba Gueye, Abdoulaye Ndoye Diop

**Affiliations:** a Department of Radiology, Hôpital de la Paix, Ziguinchor, Senegal; b UFR 2S, Gaston Berger University, Saint-Louis, Senegal; c Regional Hospital Center of Saint Louis, Senegal

**Keywords:** Fish bone migration, Cervical abscess, Computed tomography, Pediatric foreign body

## Abstract

Migration of an ingested fish bone beyond the esophageal wall is an exceptionally rare complication in children and may result in deep neck infections requiring urgent surgical management. Contrast-enhanced computed tomography (CT) plays a pivotal role in establishing the diagnosis and guiding treatment. A previously healthy 9-year-old boy presented with a progressively enlarging anterior cervical swelling associated with dysphagia and odynophagia 15 days after accidental fish bone ingestion. Clinical examination revealed a tender inflammatory cervical mass, while laboratory tests demonstrated neutrophilic leukocytosis and elevated C-reactive protein levels. Contrast-enhanced CT identified a 19-mm linear hyperdense foreign body that had migrated into the right cervical soft tissues and was surrounded by a rim-enhancing anterior cervical abscess measuring 42 × 33 × 41 mm, without vascular involvement. Surgical cervicotomy enabled complete abscess drainage and successful removal of the migrated fish bone. The postoperative course was uneventful, with complete clinical recovery. Although uncommon, transmural migration of an ingested fish bone should be considered in patients with persistent cervical symptoms following foreign body ingestion. Contrast-enhanced CT is the imaging modality of choice for accurately localizing the migrated foreign body, assessing associated complications, excluding vascular involvement, and facilitating surgical planning. Early diagnosis and prompt surgical intervention are essential to achieve favorable outcomes and prevent potentially life-threatening complications.

## Introduction

Fish bone ingestion represents one of the most common upper aerodigestive tract foreign body emergencies encountered in both otolaryngology and emergency departments [1,2]. Although the majority of ingested fish bones are successfully managed conservatively or endoscopically, transmural migration beyond the esophageal wall remains exceedingly rare and may lead to life-threatening infectious and vascular complications [3].

Contrast-enhanced computed tomography (CT) has become the imaging modality of choice for detecting migrated foreign bodies and guiding surgical management.

We report the case of a 9-year-old boy who developed an anterior cervical abscess secondary to migration of an ingested fish bone, emphasizing the pivotal role of CT in diagnosis and preoperative assessment.

## Case presentation

A 9-year-old previously healthy school-aged boy presented to our department with a progressively enlarging anterior cervical swelling that had been evolving for 15 days.

The patient's symptoms began immediately after accidental ingestion of a fish bone during a meal, resulting in persistent dysphagia and odynophagia despite several unsuccessful extraction attempts performed outside our institution.

On admission, the patient was alert and in stable general condition. Vital signs revealed a low-grade fever (38°C), tachycardia (110 beats/min), and mild tachypnea (24 breaths/min). Blood pressure was 110/70 mmHg, and body weight was 36.6 kg.

**Fig. 1:**
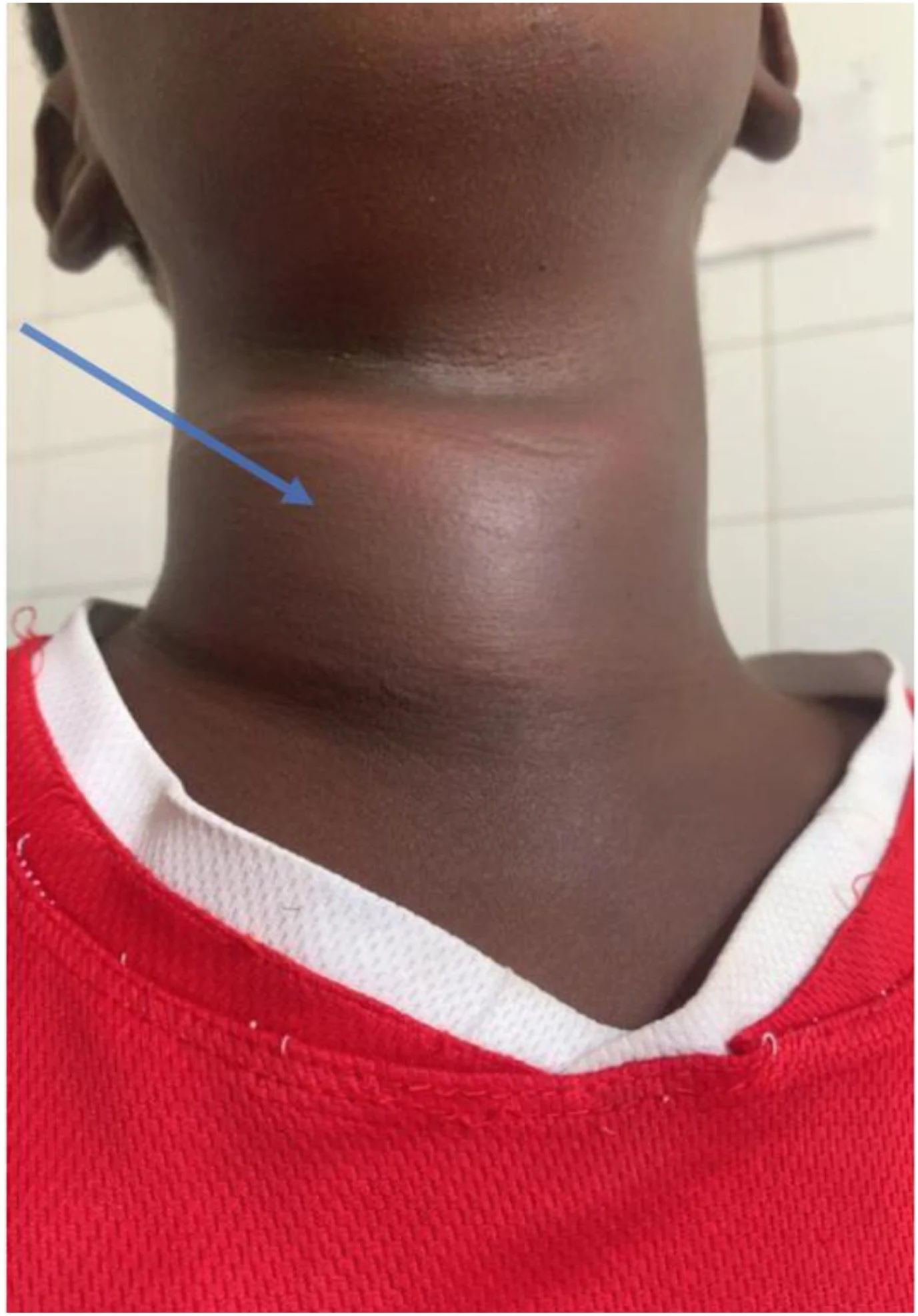
Clinical photograph demonstrating anterior cervical swelling with overlying Shiny skin (blue arrow).

Physical examination demonstrated a tender anterior cervical mass with overlying erythematous, warm, and shiny skin (Fig. 1). Otorhinolaryngological examination was otherwise unremarkable.

Laboratory investigations revealed neutrophilic leukocytosis (17,000 cells/mm^3^) and an elevated C-reactive protein level of 109 mg/L, consistent with an acute inflammatory process.

**Fig. 2:**
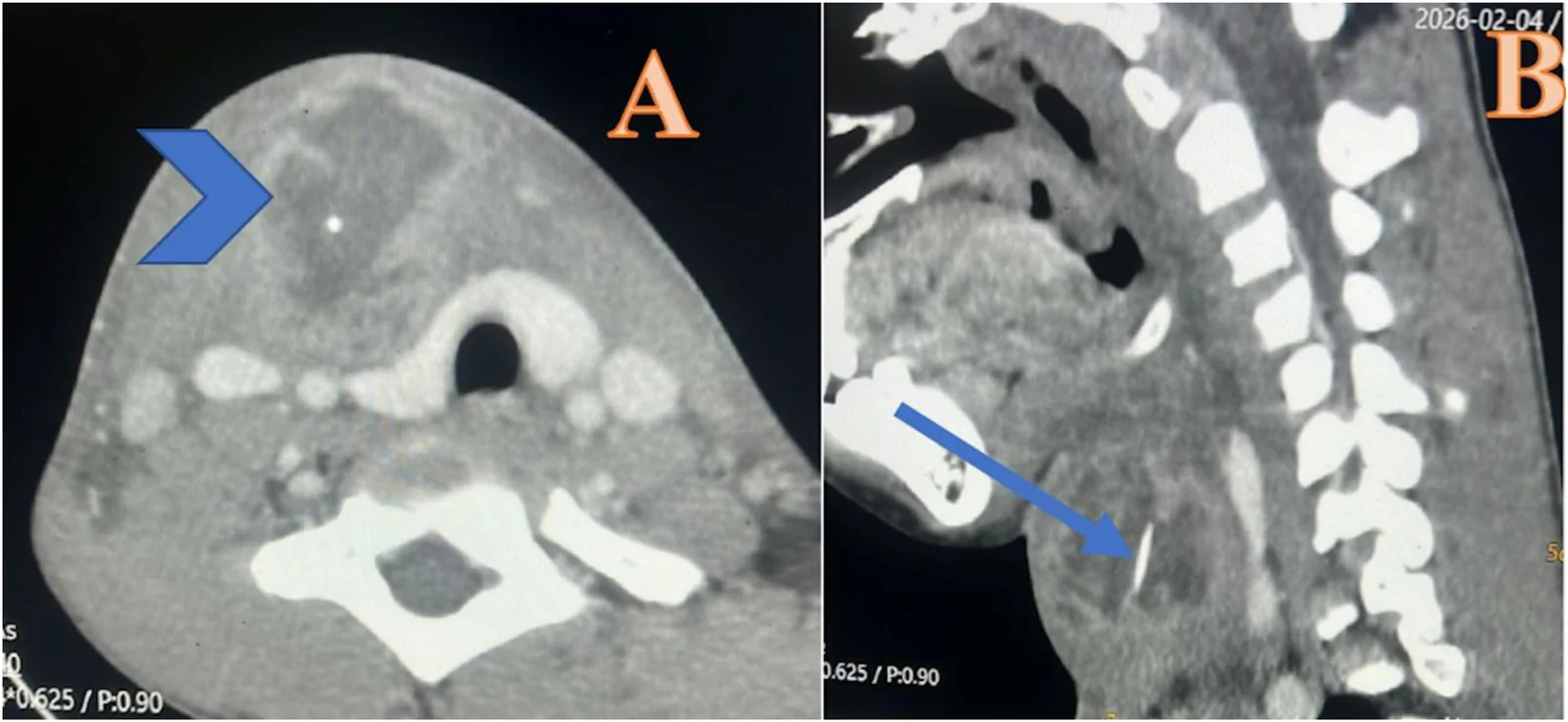
Axial (A) and sagittal reconstructed (B) contrast-enhanced CT images of the cervical region showing a hypodense fluid collection with peripheral rim enhancement (blue arrowhead), consistent with an abscess, and a linear hyperdense structure corresponding to the fish bone (blue arrow).

Contrast-enhanced cervical CT demonstrated a linear hyperdense foreign body measuring 19 mm within the right lateral cervical soft tissues. The foreign body was surrounded by a well-defined rim-enhancing fluid collection measuring 42 × 33 × 41 mm, exerting mass effect on the right thyroid compartment (Fig. 2). No vascular involvement was identified.

Based on the clinical and radiological findings, the diagnosis of an anterior cervical abscess secondary to migration of an intraesophageal fish bone was established.

**Fig. 3:**
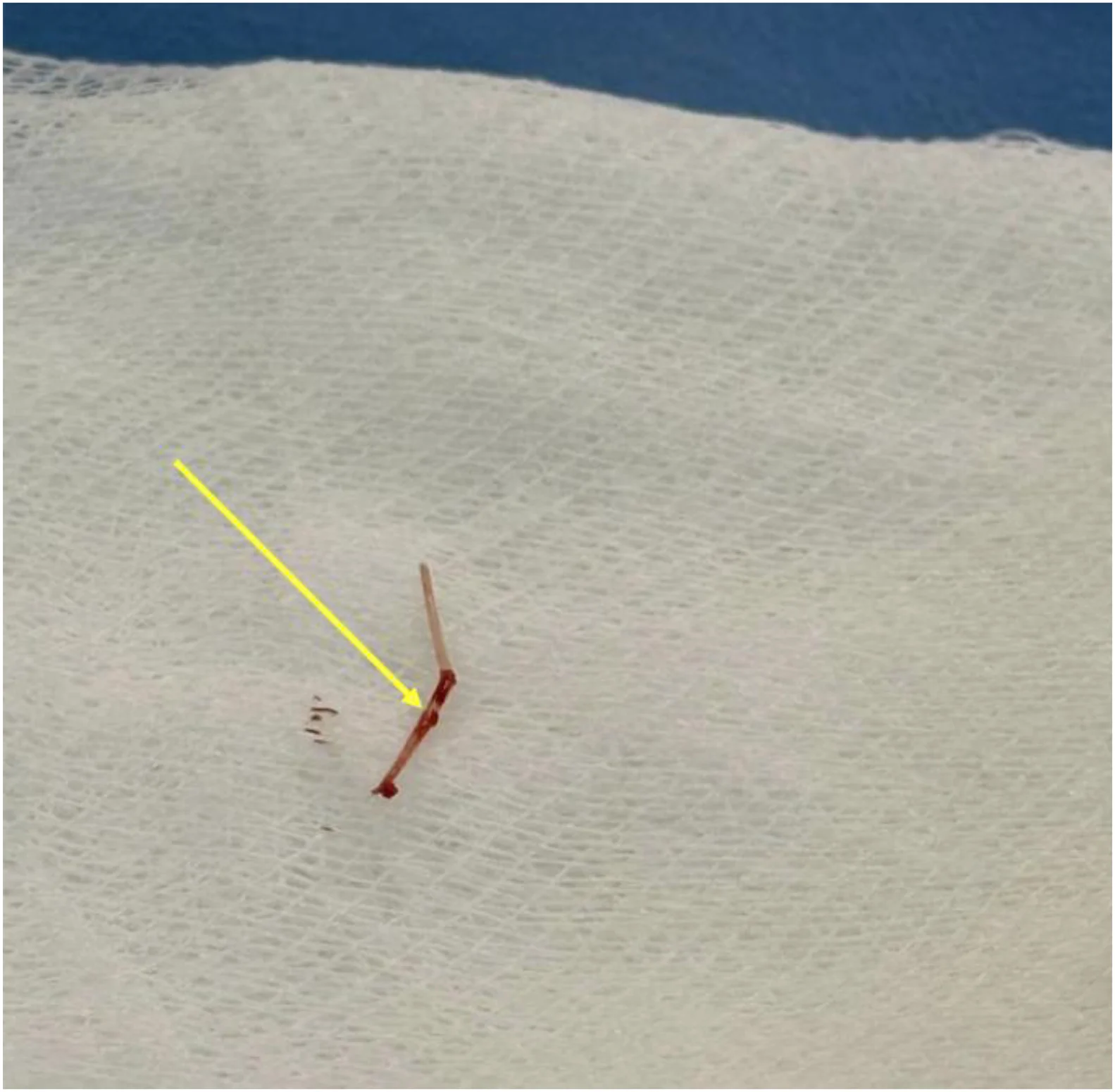
Image of the extracted fish bone (yellow arrow).

The patient underwent exploratory cervicotomy, allowing complete drainage of the abscess and successful removal of the migrated fish bone (Fig. 3).

**Fig. 4:**
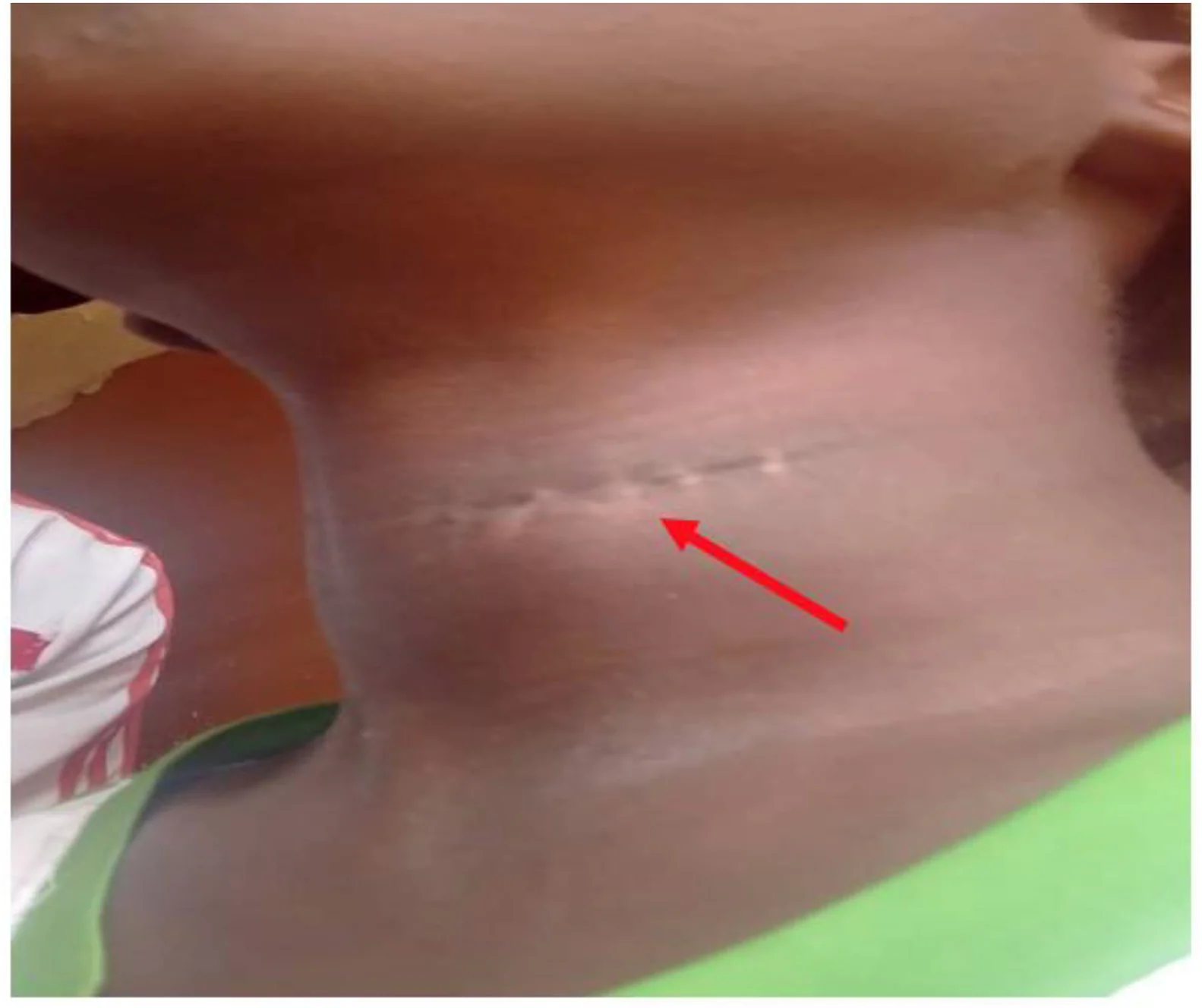
Postoperative follow-up at day 18 showing a well-healed surgical wound (red arrow) and complete resolution of the cervical swelling.

The postoperative course was uneventful. The patient received appropriate antibiotic therapy and analgesic treatment, with progressive clinical improvement. At postoperative day 18, follow-up examination demonstrated complete wound healing without evidence of recurrent infection (Fig. 4).

## Discussion

Foreign body ingestion is a common otorhinolaryngologic emergency, accounting for approximately 11% of all ENT emergency department visits Although foreign body ingestion occurs across all age groups, its epidemiology differs according to age, with fish bones being the most frequently encountered foreign bodies in adults and less common in children [4].

Most ingested foreign bodies become lodged at anatomical sites of physiological narrowing, including the palatine tonsils, valleculae, base of the tongue, and pyriform sinuses [5,6]. Approximately 80%-90% of ingested foreign bodies pass spontaneously through the gastrointestinal tract without requiring intervention. However, endoscopic removal is necessary in 10%-20% of cases, whereas surgical management is required in only about 1% of patients [7].

Many patients, aware that a fish bone is lodged in the aerodigestive tract, instinctively attempt to dislodge it by swallowing large boluses of food, rice balls, or vegetable leaves. Such practices may drive the fish bone deeper into the surrounding tissues and promote its migration to ectopic locations, thereby increasing the risk of serious complications [8].

In our setting, cervical manipulation performed by traditional healers to expel impacted fish bones also represents an important cause of fish bone migration beyond the aerodigestive tract. These external neck maneuvers may force the fish bone through the pharyngeal or esophageal wall, facilitating its migration into adjacent soft tissues and increasing the likelihood of severe infectious or vascular complications [9].

Clinical presentation is highly variable, ranging from asymptomatic cases to persistent foreign body sensation, dysphagia, odynophagia, cervical pain, and, in advanced cases, severe infectious or vascular complications [10,11].

Migration of sharp foreign bodies, particularly fish bones, represents a rare but potentially life-threatening event. Continuous swallowing movements and local inflammatory reactions may facilitate progressive penetration through the esophageal wall into the surrounding cervical soft tissues. Once migration has occurred, secondary bacterial contamination may lead to deep neck infections, abscess formation, mediastinitis, thyroid involvement, or even major vascular injuries involving the carotid artery or jugular vein.

In the present case, migration of the fish bone resulted in the formation of an anterior cervical abscess without vascular involvement. A similar presentation has previously been reported by Elansari et al. [4], emphasizing the rarity of this complication in pediatric patients [4].

Cross-sectional imaging is indispensable when migration of an ingested foreign body is suspected. Among available imaging modalities, contrast-enhanced computed tomography is considered the diagnostic modality of choice because of its excellent sensitivity for detecting radiopaque foreign bodies, accurately defining their location, assessing their relationship to adjacent anatomical structures, and identifying associated complications such as abscess formation or vascular injury [9]. In our patient, CT successfully demonstrated the migrated fish bone, characterized the surrounding abscess, excluded vascular involvement, and provided essential information for preoperative surgical planning.

Computed tomography (CT) has a detection rate approaching 100% for ingested fish bones [12]. It is a noninvasive imaging modality that combines thick-section acquisition with thin-section and 3-dimensional (3D) reconstructions. This approach enables accurate identification of fish bones and precise localization. Assessment of the size and morphology of the fish bone, as well as associated complications such as abscesses, hematomas, and other adjacent lesions, is essential for guiding appropriate clinical management.

Chen et al. [13] demonstrated that contrast-enhanced CT combined with multiplanar reconstruction (MPR) significantly improves the detection of ectopic fish bones and their secondary complications, supporting its use as the first-line imaging modality in patients with suspected fish bone migration [13].

However, Shishido et al. [14] highlighted several limitations of CT. Its sensitivity decreases for poorly calcified or noncalcified fish bones, while normal anatomical structures may mimic foreign bodies and lead to false-positive interpretations. In addition, deeply embedded fragments may be difficult to detect, and variations in fish bone morphology among different fish species, together with indirect imaging findings, may further complicate image interpretation [14].

Overall, CT is markedly superior to conventional radiography and contrast esophagography for the detection of fish bones and should be considered the imaging modality of choice in patients with suspected fish bone impaction or migration.

Once migration has occurred, conservative or endoscopic management is generally no longer appropriate. Surgical exploration through cervicotomy remains the treatment of choice, allowing complete drainage of infected collections and safe removal of the migrated foreign body while minimizing the risk of recurrent infection or injury to adjacent neurovascular structures [9].

When diagnosis and surgical management are performed promptly, the prognosis is generally favorable, with low postoperative morbidity and excellent functional outcomes, as illustrated by the uneventful recovery observed in our patient [4].

## Conclusion

Fish bone ingestion is a common clinical condition, particularly among adults, although it may also occur in children. While most ingested fish bones pass spontaneously or are successfully removed endoscopically, migration beyond the esophageal lumen is an uncommon but potentially serious complication.

This case illustrates that migration of a fish bone may result in deep cervical infection with abscess formation, even in pediatric patients. Early recognition of this rare complication is essential to prevent potentially life-threatening infectious and vascular sequelae.

Contrast-enhanced computed tomography plays a central role in establishing the diagnosis, accurately localizing the migrated foreign body, evaluating its relationship with adjacent structures, identifying associated complications, and guiding surgical management.

Prompt surgical intervention combined with appropriate antimicrobial therapy remains the cornerstone of treatment and is associated with an excellent prognosis when performed without delay.

## Patient consent

Written informed consent for the publication of this case report was obtained from the parents.
